# Evaluation of the effect of Remdesivir on some biomarkers in Iraqi patients with coronavirus 2019 (COVID-19): A cross-sectional study

**DOI:** 10.25122/jml-2023-0209

**Published:** 2023-08

**Authors:** Maysaa Ali Abdul Khaleq

**Affiliations:** 1 Department of Pharmacy, AL-Maarif University College, AL-Anbar, Iraq

**Keywords:** COVID-19, ferritin, inflammatory protein C, lactate dehydrogenase, RDV: Injectable antiviral Remdesivir, FDA: Food and Drug Administration, CRP: C-reactive protein, LDH: Lactate Dehydrogenase, CTLs: Cytotoxic T lymphocytes, NK: Natural Killer

## Abstract

COVID-19 is a new virus spreading worldwide that can cause mild to severe illness, multi-organ failure, and even death. Injectable antiviral Remdesivir is effective in treating patients with moderate-to-severe COVID-19. Biomarkers linked to clinical outcomes have been found for COVID-19, although only a few antiviral therapies have been studied. This study aimed to assess how Remdesivir affects several biomarkers in patients with COVID-19 and how those changes impact the severity of the illness. According to Chinese care guidelines for COVID-19, 80 patients with COVID-19 were separated into two groups: group 1 did not receive Remdesivir (RDV) medication and Group 2 received it after 5 days. Injectable antiviral Remdesivir has recently been tested in high-risk, individuals with confirmed SARS-CoV-2 infection who were not hospitalized, and it successfully delayed the onset of the illness. From February 2022 to October 2023, blood samples were taken from study participants to evaluate ferritin, Lactate Dehydrogenase (LDH), and C-reactive protein. The results of this investigation showed that various COVID-19 severity biomarkers, including ferritin, C-reactive protein, and lactate dehydrogenase, may improve more quickly with RDV treatment. These biomarkers are linked to better clinical outcomes during infection. These discoveries enhance the understanding of the COVID-19 antiviral treatment’s function. In conclusion, there is a clear association between the levels of biomarkers before and after Remdesivir treatment in COVID-19 cases ranging from moderate to severe. This suggests that the COVID-19 infection might lead to the elevation of several biomarkers.

## INTRODUCTION

The novel human coronavirus, SARS-CoV-2, has spread worldwide at an unprecedented rate since its initial emergence in December 2019. Following its phylogenetic similarity to SARS-CoV-1, the virus was named SARS-CoV-2 by the International Committee on Taxonomy of Viruses [1]. In the Middle East and East Asia, significant disease epidemics have been connected to coronaviruses. In 2002 and 2012, respectively, the first instances of Middle Eastern Respiratory Syndrome (MERS) and severe acute respiratory syndrome (SARS) were reported [2]. Today, a novel coronavirus is responsible for the 2019 Coronavirus Disease (COVID-19), which emerged in 2019 and spread continuously over many nations and territories, posing a threat to global health [3]. COVID-19 belongs to a group of viruses named coronaviruses [4]. The four primary subgroups are alpha, beta, gamma, and delta. According to research, seven different types of coronaviruses can infect humans, including the human coronavirus 229E (HCoV-229E), human coronavirus NL63 (HCoV-NL63), beta coronaviruses, and the recently identified COVID-19, causing SARS-CoV2 [4]. Remdesivir is a nucleotide prodrug analog of adenosine. By prematurely terminating ribonucleic acid (RNA) transcription when binding to the viral RNA-dependent RNA polymerase, it prevents viral replication. Remdesivir has demonstrated in vitro and in vivo activity against SARS-CoV-2 [5]. In vitro, Remdesivir neutralizes the Omicron variant and its sub-variants [6]. The United States Food and Drug Administration (FDA) permits the administration of intravenous Remdesivir for treating COVID-19 in adults and children at least 28 days old and weighing more than 3 kg. For out-patients with mild-to-moderate COVID-19 at high risk of developing severe disease, Remdesivir should be administered for three days, at seven days following the onset of the first symptoms. In-patients should take Remdesivir for 5 days or until discharged, whichever comes first [7]. The paper aims to assess how Remdesivir affected several biomarkers in patients with COVID-19 and how those changes impact the severity of the illness.

### Laboratory diagnosis and SARS-COV-2 (essential laboratory parameter)

A monomeric acute-phase inflammatory protein, C-reactive protein (CRP) was named after interacting with Pneumococcus capsular (C)-polysaccharide [8]. It can be found in various vertebrate and invertebrate species and belongs to the same family as serum amyloid a [9], which also comprises additional compounds with structurally similar components. The CRP gene is primarily activated in hepatocytes in response to escalating levels of inflammatory cytokines, particularly interleukin-6 (IL-6). The concentration of CRP increases due to inflammatory conditions, such as infections, rheumatoid arthritis, and various heart illnesses [10]. As the first innate defensive mechanism in response to the inflammatory onset, CRP attaches to pathogens and assists in their removal by phagocytic cells. CRP in the host defense mechanism during infection. By preventing neutrophil chemotaxis, CRP can also have anti-inflammatory effects [11]. Contrarily, CRP can promote adhesion molecule production, including inflammatory substances, such as Interleukin 1 (IL-1), IL-6, IL-8, and tumor necrosis factor alfa (TNF-a) [12].

### Lactate dehydrogenase (LDH)

A hydrogen transfer enzyme, lactate dehydrogenase (LDH), uses NAD+ as a hydrogen acceptor, thus accelerating the reversible oxidation of L-lactate to pyruvate. The two types of 4-peptide chains that made up lactate dehydrogenase-M (type A) and H (type B) were genetically controlled individually [13]. Many cells throughout the body had lactate dehydrogenase activity, and different tissues had enzyme concentrations 1,500–5,000 times higher than those observed in the blood. Therefore, even a small amount of damaged tissue leaking the enzyme causes a large increase in the observed blood activity of LDH [14]. Elevated LDH is a sign of poor outcomes in immunosuppressed people [15]. In addition to boosting immune-suppressive cells, like dendritic cells (DCs) and macrophages, LDH also enhances lactate synthesis and suppresses cytolysis cells, such as cytotoxic T lymphocytes (CTLs) and natural killer (NK) cells [16].

### Ferritin

Ferritin is a protein that binds iron found in extracellular and intracellular compartments. Elevated serum ferritin has been suggested as a predictive factor for COVID-19 mortality [17]. A fifth affection associated with hyperferritinemia, except for the four known clinical entities (adult-onset Still’s disease, catastrophic antiphospholipid syndrome, macrophage activation syndrome, and septic shock) may be COVID-19, according to clinical and laboratory findings. These disorders share a severely increased blood ferritin level and a potentially fatal hyperinflammation [18]. Following COVID-19 infection, ferritin may contribute to inflammation. Macrophages produce active ferritin, and cytokines can lead to hyperferritinaemia, leading to the production of several cytokines, pro- and anti-inflammatory, such as IL-1, IL-2, and IL-10 [19].

## MATERIAL AND METHODS

This cross-sectional research included 80 COVID-19 patients (31 female and 49 male). The participants were venipunctured with a syringe to obtain five milliliters of blood. The blood samples were taken from the upper limb’s superficial vein. Some biomarkers, including ferritin, LDH, and CRP, were measured using an auto-analyzer (spin 200E analyzer), and the measurements between the two groups were compared.

### Statistical analysis

The data was analyzed using the Statistical Package for Social Sciences (SPSS) version 19.0 and Microsoft Excel 2013. The categorical data are expressed using counts and percentages. The correlation between these variables was examined using the Chi-square test. If there were 25% fewer cells than expected, the Fisher exact test was applied. The mean, standard error of the mean, and standard deviation were used to characterize numerical data. The t-test for independent samples was used to compare two groups, and the analysis of variance (ANOVA) was performed for parametric data. Correlation coefficient tests or the Pearson equation between variables were used. The alternative theory was evaluated using the Pearson equation among the variables or correlation coefficient tests. The null hypothesis was tested, and the P-values were used to assess the significance of the testing. Results were considered significant at a P-value of less than 0.05.

## RESULTS

In this cross-sectional investigation, 80 patients with COVID-19 received a clinical diagnosis and had coronavirus RNA detected by reverse transcriptase polymerase chain reaction (RT-PCR) with a positive result. The patients were split into two groups: group 1 did not receive Remdesivir treatment, and group 2 received Remdesivir treatment. Between the two study groups, there was a noticeable distinction in measured values of ferritin and CRP. Ferritin and CRP in group 1 had a mean ± SD of 707.078±552.234, and 99.460±67.641, respectively, while in group 2, the mean ± SD values for ferritin and CRP were 206.411±161.050 and 33.481±33.671. Although there was no statistically significant difference between the groups, the estimated level of LDH was elevated in all patients. According to Table 1, the means and standard deviations of LDH in groups (1) and (2) were 737.074±364.913, and 626.231±365.740, respectively.

**Table 1:** Estimation of Ferritin, C-reactive protein, and Lactate dehydrogenase in COVID-19-positive patients

Parameter	Groups	Mean ± Std. Deviation	p-value
Ferritin (µg/L)	Before treatment with Remdesivir (group 1)	707.078±552.234	0.000*
	After treatment with Remdesivir (group 2)	206.411±161.050	
CRP (mg/L)	Before treatment with Remdesivir (group 1)	99.460±67.641	0.000*
	After treatment with Remdesivir (group 2)	33.481±33.671	
LDH (units/L)	Before treatment with Remdesivir (group 1)	737.074±364.913	0.174
	After treatment with Remdesivir (group 2)	626.231±365.740	

* p<0.05

The results of the current investigation showed no correlation between ferritin and LDH before or after therapy, but a substantial correlation between ferritin and CRP. Table 1 displays the precision of the ferritin and CRP tests in the diagnosis and therapeutic response in COVID-19-infected patients.

## DISCUSSION

A novel coronavirus has been identified in humans causing COVID-19. Most patients with COVID-19 face mild to moderate pulmonary symptoms. However, more severe forms may manifest in the elderly and people with underlying medical conditions, such as cancer, diabetes, cardiovascular disease, or persistent respiratory infections. According to recent studies, the inflammatory process causes patients with COVID-19 to have greater ferritin levels. Today, doctors utilize hyperferritinemia as a marker for an acute-phase reaction to assess patients’ responses to treatment. Contrarily, recent studies show that ferritin levels can increase during the acute-phase reaction and play a crucial part in the inflammatory response following a cytokine storm [20]. Ferritin is an iron-storing protein that can also store calcium intracellularly. The liver, spleen, and bone marrow all contain the ferritin necessary for hematopoiesis and iron recycling. Serum, plasma, and erythrocytes can have ferritin [21]. Remdesivir (RDV), a nucleoside analog, inhibits the reproduction of the SARS-CoV-2 virus by binding to the viral RNA-dependent RNA polymerase. Clinical trials with RDV revealed that in out-patients at risk for COVID-19, a 3-day course of the medication might reduce such risks by 87% [22]. Research on COVID-19 patients demonstrated that those who received RDV recovered more quickly than those who received a placebo (10 days *vs*. 5 days, respectively), and they were also released from the hospital sooner (12 days *vs*. 17 days, respectively) [23]. The COVID-19 group participating in this study’s five-day therapy period demonstrated a statistically significant difference in the measured value of ferritin. Ferritin had a 351.00 g/L cutoff value, with 70% sensitivity and 20% specificity. When all of these findings were considered, it was discovered that serum ferritin levels were much greater before therapy in COVID-19 patients, and hyperferritinaemia was both an independent risk factor and a predictor of disease severity. There are two methods to explain ferritin’s importance. According to Shoenfeld *et al*., the macrophage activation syndrome, marked by high ferritin levels and a cytokine storm, is similar to the clinical course of patients with severe COVID-19. When macrophages are activated by ferritin’s H-chain, a cytokine storm and increased ferritin levels result. In patients, the H-chain of ferritin stimulates macrophages and causes them to release more inflammatory cytokines [24]. According to American research, people with COVID-19 had decreased hemoglobin levels and greater ferritin concentrations in their blood. In 5,700 patients admitted to the hospital with COVID-19, ferritin levels were found to be excessively high [25]. Anemia brought by hyperferritin is a potent predictor of mortality, regardless of the underlying illnesses. Iron metabolism may be impacted by increased ferritin levels in COVID-19, which may be a marker of an oncoming inflammatory response or related to viral spread in the body [26]. This study’s findings were consistent with those of Bozkurt *et al*. (2021) and Beigel *et al*. (2020), who found that the level of ferritin in severely ill patients was much higher before treatment [27-28]. CRP, a metameric protein, is produced by the liver during the inflammatory/infectious process for the acute-phase reactant protein by the action of IL-6 on the CRP transcription gene. CRP has pro- and anti-inflammatory actions. By adhering to phosphor choline, phospholipids, histone, chromatin, and fibronectin, it aids in identifying and removing invasive infections and injured cells [29]. After the commencement of the disease, CRP level rises swiftly (6–8 hours) and peaks 48 hours later despite having a normal blood concentration of less than 10 mg/l. Its concentration decreases as the patient heals and the inflammatory stages pass since it has a half-life of about 19 hours. CRP tends to bind to phosphor choline, which is extensively produced on the surface of injured cells. This binding triggers the immune system’s classical complement pathway and controls phagocytic activity, which rids the body of pathogens and damaged cells. High CRP levels may be linked to an overproduction of inflammatory cytokines in COVID-19 individuals with severe symptoms. Patients with COVID-19 have CRP production as a result of tissue damage and inflammatory cytokines [30]. The current study’s findings supported research by Guan WJ *et al*. (2020) and Goldman JD *et al*. (2020) highlighting an increased level of CRP in severe cases, before Remdesivir treatment [31-32]. These studies also found that patients with COVID-19 had significantly higher CRP levels before and after treatment. Several biomarkers are being researched for their possible role in determining prognosis in COVID-19 patients. Lactate dehydrogenase is another important biomarker, in particular, because higher LDH levels have in the past been linked to worse outcomes in people with a variety of viral diseases [33]. Early research in COVID-19 patients points to a considerable variation in LDH concentrations between those with severe disease and those without [34]. Contrary to earlier research, the current study’s findings indicated no significant difference in LDH levels before and after treatment [35].

## CONCLUSION

A considerable rise in ferritin, CRP, and lactate dehydrogenase indicates an inflammatory response in COVID-19 patients before antiviral therapy. Remdesivir (RDV) is a nucleoside analog that binds to the viral RNA-dependent RNA polymerase, the enzyme that makes copies of the viral RNA and inhibits SARS-CoV-2 viral replication to decrease the inflammatory biomarkers associated with COVID-19 patients. Studies in patients with COVID-19 found that those who received RDV recovered faster than those who had not. Further research should evaluate various other biomarkers in COVID-19 patients. The accurate evaluation of laboratory indicators at the start of COVID-19 and throughout can help doctors prescribe additional treatments that are appropriate and provide timely, specialized care to patients.
